# Chromosome-level genome assembly of the common tenrec, *Tenrec ecaudatus* (Schreber, 1778), a new model for early placental mammal evolution

**DOI:** 10.1186/s12864-026-12794-9

**Published:** 2026-03-31

**Authors:** Damián Hernández-Roco, Evgeny Leushkin, Jacqueline Galeas, Katharine R. Grabek, Carlos Lopez, Gilbecca Rae Smith, Daniel J. Stadtmauer, Günter P. Wagner, Frank van Breukelen, Michael Hiller, Linda Goodman, Patrick Arnold

**Affiliations:** 1 Evolutionary Adaptive Genomics, Institute of Biochemistry and Biology, University Potsdam, 14476 Potsdam, Germany; 2 Senckenberg Research Institute, Senckenberganlage 25, 60325 Frankfurt, Germany; 3 Fauna Bio, Inc, Emeryville, CA 94608 USA; 4 School of Life Sciences, University of Nevada, Las Vegas, NV 89154 USA; 5 Department of Genetics, Harvard Medical School, Boston, MA 02115 USA; 6 Department of Ecology & Evolutionary Biology, Yale University, New Haven, CT 06511 USA; 7 Department of Evolutionary Biology, University of Vienna, Vienna, 1030 Austria; 8 Institute of Cell Biology and Neuroscience, Faculty of Biosciences, Goethe University Frankfurt, Max-von-Laue-Str. 9, 60438 Frankfurt, Germany

**Keywords:** Tenrec, Genomics, Afrotheria, Afroinsectiphilia, Madagascar, Phylogenetics, Synteny, Chromosome-level, Genome

## Abstract

**Background:**

Our understanding of many biological aspects of early placental mammals is still very limited. Due to the paucity of the fossil record, attention is often turned to those extant organisms that share plesiomorphic characters in order to gain insights into the evolutionary history of mammals. Afrotheria is one of the four major clades of placental mammals, accounting for around a third of all mammalian orders, and encompasses a wide array of differently adapted species. Within Afrotheria, tenrecs are a more species-rich group of small mammals native to the island of Madagascar that display several special traits resembling those hypothesized on early placentals regarding reproductive strategies, thermoregulation and growth metabolism. Despite this, tenrecs remain heavily understudied in many aspects. Genomic information for this group of mammals is scarce and not up to modern quality standards.

**Results:**

We present here the complete, chromosome-scale reference genome and annotation of the common tenrec, *Tenrec ecaudatus*. To put this new resource to use, we conducted a phylogenetic reconstruction and divergence time estimation for Afrotheria using all the available genomic resources for afrotherian mammals. This analysis recovered the phylogenetic order containing hyraxes as a sister group to elephants and a younger molecular divergence of tenrecs than previously estimated. Added to this, our comparative chromosome-synteny analyses showed significant rearrangements within afrotherians, especially on the clade shared by tenrecs, elephant-shews and the aardvark (Afroinsectiphilia).

**Conclusion:**

This newly produced high-quality genome assembly proves to be a valuable resource to complement our genomic understanding of Afrotheria, allowing for insights into chromosome evolution, time of molecular divergence and phylogenetic reconstruction. This establishes a basis for further studies to utilize this resource to further pursue evolutionary questions regarding tenrecs adaptations and comparative analyses within Afrotheria.

**Supplementary Information:**

The online version contains supplementary material available at 10.1186/s12864-026-12794-9.

## Background

Afrotheria is one of the four major placental mammal clades, comprising around one third of its orders [1]. This taxon unites ungulate-like (Paenungulata: elephants, sea cows, hyraxes) and insect-eating lineages (Afroinsectiphilia: aardvarks, sengis, tenrecs, golden moles). Despite displaying this diverse array of morphological features and ecological specializations that have sparked debate about the validity of the clade [2–4], this group has been categorized as monophyletic by numerous taxonomic assessments employing modern molecular methods [5–8]. Afrotherians, as their name suggests, are hypothesized to have originated on the African continent between around 100 million years ago (Mya) and later highly diversified during the late Cretaceous period when the South American landmass fully separated from the rest of Africa, leaving it isolated [9–11].

However, not all afrotherian species remained confined within continental Africa. Such is the case of the afrosoricidan (tenrecs and golden moles) family Tenrecidae, an insectivoran-grade lineage that colonized the island of Madagascar in the Indian ocean between 56 and 30 Mya [12] and subsequently radiated into the species-rich yet understudied group of mammals [13, 14]. Commonly known as ‘Malagasy tenrecs’, this group of small mammals includes 31 currently accepted extant species [15], with four species classified as Vulnerable (VU) and two species classified as Endangered (EN) by the International Union for Conservation of Nature (IUCN). Yet, the number of threatened species is expected to increase in the following decade [16]. In addition, alpha taxonomy of tenrecs is still not fully understood and the cryptic diversity might be higher [17, 18]. Although the phylogenetic relationships among major tenrec lineages are relatively well established, the dates of divergence among them are less clear due to the highly limited molecular resources available for tenrecids so far [12].

This relatively unknown group of mammals displays several unique characteristics that makes them worthy of broader scientific interest [19]. For example, tenrecs retain reproductive flexibility that likely mirrors early placental mammals [20]. Unlike most modern placentals, they exhibit superfetation, polyovulation, large litter sizes, and a lack of traditional antrum formation, suggesting a reproductive strategy that prioritized offspring quantity over metabolic efficiency, which may reflect strategies employed by early placental mammals [21–24]. Indeterminate growth is also considered the ancestral trait of amniotes [25] and it’s rarely documented in mammals. Tenrecs vary widely in size at birth and exhibit striking developmental variability.

**Fig. 1:**
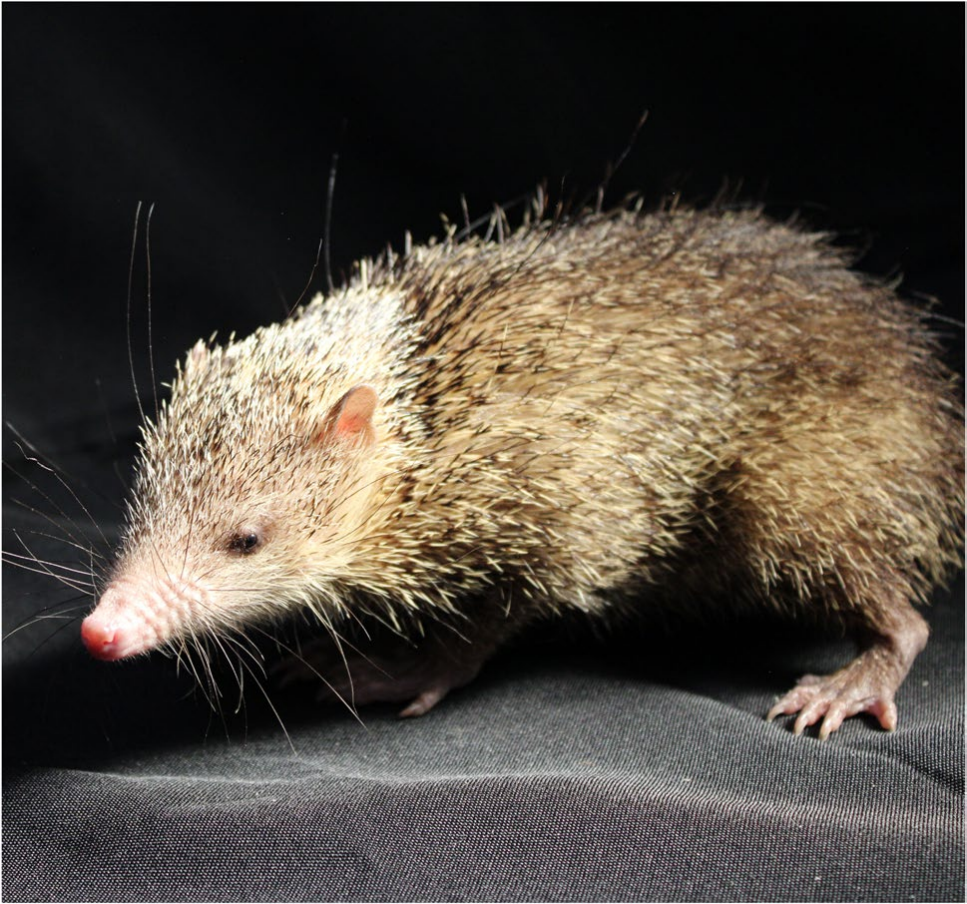
Individual of *T. ecaudatus* from the breeding colony at the university of Nevada, Las Vegas, USA. photograph by Sophia Lorenzana

*Tenrec ecaudatus* (Schreber, 1778), also known by the common name ‘tailless tenrec’ or ‘common tenrec’ (Fig. 1), is largest of all extant tenrecs and the only species of tenrec to have been introduced through human activity to several islands along the Mascarenhas archipelago, the Comoros and the Seychelles [19, 26]. Tailless tenrecs being such a widespread species in between islands has resulted in the existence of multiple reports of individuals acting as a reservoir of *Leptospira* and causing several zoonotic outbreaks [27–29]. This added to the fact that tenrecs are an important part of the bushmeat diet of Malagasy households [30–32], makes *T. ecaudatus* a species of special human interest. Among other particularities of this species, *T. ecaudatus* has remarkable thermal plasticity. [24].

Taking into account all the peculiarities of Tenrecidae in general and the human relevance of the species, *T. ecaudatus* makes a suitable candidate to be a model organism for physiological, developmental and evolutionary research on tenrecs, afrotherians and placental mammals as a whole [33–35]. Increased availability of chromosome-scale genome assemblies and high-throughput sequencing technology has made it both possible and almost a requirement to generate high-quality genomic data for non-model species [36–39]. Chromosome-scale genome assemblies also enable investigating the chromosome evolution and conservation across afrotherian lineages. Notably, afrotherians have been shown to display significant variation in chromosome number not only among but also within orders [40, 41]. Chromosome synteny between some members of Afrotheria has so far been approached by chromosome painting [41–43], but no real genome-level comparison of all orders has been conducted so far.

In this study, we generated a haplotype-resolved, chromosome-level genome assembly for the tailless tenrec, *T. ecaudatus*. Furthermore, we supplemented this genomic resource with orthology-based annotation, phylogenomic and dating analysis as well as chromosome synteny. As a result, this genomic resource makes possible further research into Tenrecidae from a genomic standpoint, allowing for insight into previously unknown aspects of this group’s biology and evolutionary history.

## Materials and methods

### Sample collection

Samples were taken from lab-reared common tenrec individuals from the breeding colony maintained at the University of Nevada, Las Vegas (UNLV), USA (Fig. 1). The breeding colony originated from 40 wild-caught individuals imported from Mauritius in June 2014 under appropriate U.S. federal and state permits. Laboratory-reared tenrecs of male and female sex (Table S1) were euthanized via cervical dislocation followed by pneumothorax, and immediately dissected on ice. Heart, brain, kidney, testis, liver and embryotic tissue was extracted, rinsed in diethyl pyrocarbonate (DEPC)-treated water, and snap-frozen in liquid nitrogen. Samples were stored at − 80 °C until sent to FaunaBio (Emeryville, CA, U.S.) for further processing. In addition, liver, kidney, uterus, and placenta were collected as above from a pregnant female reported in [20], preserved in RNAlater (AM7021, Thermo Fisher) and shipped to Yale University (New Haven, CT, USA) for processing.

### DNA/RNA extraction and pacBio sequencing

Heart, brain, kidney and liver samples underwent liquid nitrogen pulverization and DNA was then extracted using the Monarch HMW DNA extraction kit for tissue (New England Biolabs) in accordance with protocol instructions. Liver and heart tissue from individual #F20376 (Table S1) were used for PacBio sequencing. To process the liver sample, NaCl was utilized to remove polysaccharides and avoid glycogen contamination. Final quality and concentration measuring of DNA was performed with NanoDrop, Qubit Fluorometer and Femto quantification. Library preparation was carried out using the SMRTbell prep kit 3.0 (Pacific Biosciences, Menlo Park, CA). The library was sequenced on a PacBio Sequel II using the Sequel II binding kit 3.2 at QB3 Genomics, (UC Berkeley, Berkeley, CA). While aiming for 50x coverage, a total of 9 flow cells were run. Additionally, at this same facility PacBio Iso-Seq data was generated for liver, kidney, brain, testes, heart and embryonic tissue using 1,250 ng/sample. RNAlater-preserved samples from a pregnant female were extracted using a Qiagen RNEasy Plus Micro Kit (Q74034) and sequenced on a PacBio Sequel II at the Yale Center for Genome Analysis.

### Hi-C chromatin conformation capture

Chromatin conformation capture was carried out in the facilities of Arima Genomics, Carlsbad, USA using the ARIMA-HiC kit for animal tissue (Document Part Number: A160132 v01) on heart and liver tissue from individual #F20376 and following protocol’s guidelines. Illumina library preparation was performed following the ARIMA Library Prep Module user guide (Document Part Number: A160432 v02). The barcoded HiC library was then sequenced on an Illumina NovaSeq6000 platform with 150 bp paired-end reads.

### Genome assembly & annotation

Prior to the assembly, we called HiFi reads from PacBio subreads using pbccs v.6.4.0 (https://github.com/nlhepler/pbccs) and deepconsensus v.1.1.0 [44]. HiFi reads were assembled using hifiasm v.0.18.7 [45], with Hi-C reads used for phasing of the two haplotypes. We then mapped HiFi reads back to each assembly with minimap2 v.2.24 [46] to remove unambiguous heterozygous sites, removed duplicates using Picard MarkDuplicates tool v.3.1.0 (http://broadinstitute.github.io/picard), and called variants using DeepVariant v1.5.0 [47]. Finally, we corrected corresponding nucleotide sites in the assembly using BCFtools consensus v.1.13 [48].

To scaffold the polished haplotype assemblies, we mapped the Hi-C reads to each of the two assemblies using Chromap v.0.2.5 [49] and then processed mapped reads with yahs v1.1a [50]. We then concatenated the two yahs-scaffolded assemblies and remapped the Hi-C reads but now allowing for multimapping reads (-q 0) to avoid losing reads, mapped to regions identical between two haplotypes. This allowed us to cross-validate two haplotypes by manually curating them in parallel in PretextView v.0.2.5 (https://github.com/wtsi-hpag/PretextView). Additionally, telomeric sequences were identified with tidk v.0.2.31 (https://github.com/tolkit/telomeric-identifier) and where necessary were used to correct wrong contig orientations to have telomeres in the ends of resulting scaffolds. To finalize changes made via manual curation, as recently proposed by the Vertebrate Genomes and Darwin Tree of Life Projects, we used the rapid curation framework (https://gitlab.com/wtsi-grit/rapid-curation/-/tree/main) from the Genome Reference Informatics Team [51]. To estimate the assembly base accuracy, we used Merqury [52] with the HiFi reads. To assess gene completeness, we used compleasm 0.2.6 [53] with the BUSCO (Benchmarking universal single-copy orthologues) odb10 set of 9226 near-universally conserved mammalian genes.

We used a combination of orthology inference and transcriptome sequencing to produce whole-genome annotations. For each of the two haplotype assemblies we generated alignment chains to human (hg38) as described previously [54, 55] and then used TOGA v.1.1.4 [56] to annotate coding genes with human GENCODE 38 annotation serving as reference. We then processed previously produced Iso-Seq reads to obtain transcriptomic evidence for each tissue separately. Circular consensus was generated from the subreads using ccs v.6.4.0 (https://github.com/PacificBiosciences/pbbioconda). Adapter trimming and base quality filtering were performed with lima v2.2.0 (https://github.com/PacificBiosciences/pbbioconda). Processed reads were cleaned from polyA tails and polymerase switching artifacts using “refine” command from the Iso-Seq package v.4.0.0 (https://github.com/PacificBiosciences/IsoSeq/blob/master/isoseq-clustering.md) and then clustered together and aligned to generate consensus reads using the “cluster” command from the Iso-Seq package. Additionally, reads were filtered for alignment quality using a custom Perl script (https://github.com/hillerlab/GenomeAlignmentTools/blob/master/src/filterMinimapQuality.perl*).* Finally, StringTie v.2.1.2 [57] was used to produce assembled transcripts. Individual tissue annotations were then merged together. Finally, in cases where TOGA prediction was missing, transcriptome evidence was used as a primary annotation source, incorporating for the most part lineage-specific and noncoding transcripts. Comparative quality metrics with the additional genome assemblies included in the study were latter assessed with Quast v5.2 [58], BBMap v37.62 [59] and Compleasm v0.2.6 [53].

### Phylogenomic analyses

For the phylogenetic reconstruction of Afrotheria, we first downloaded full genome annotation data already available at the time, focusing on Tenrecidae and adding representatives from all remaining afrotherian clades. We assembled a dataset obtaining human reference-annotated data from the TOGA annotations for mammals repository on the Senckenberg Genome Browser (https://genome.senckenberg.de/TOGA.mammals.html). For species of interest without an available annotation on this database, we supplemented it by producing our own annotation for available afrotherian genome assemblies also using the make_lastz_chains (https://github.com/hillerlab/make_lastz_chains*)* and TOGA v1.1.4 [56] pipeline. This resulted in the creation of a dataset comprising 14 afrotherian species (Table S2), including our own *Tenrec ecaudatus* annotation data. This dataset encompassed sea cows (Sirenia: *Dugong dugon*, *Trichechus manatus*), elephants (Proboscidea: *Loxodonta africana*, *Elephas maximus*), hyraxes (Hyracoidea: *Heterohyrax brucei*, *Procavia capensis*), the aardvark (Tubulidentata: *Orycteropus afer*), a golden mole (Chrysochloridae: *Chrysochloris asiatica*), sengis (Macroscelidea: *Rhynchocyon cirnei*, *Elephantulus edwardii*, *Petrodromus tetradactylus*) and tenrecs (Tenrecidae: *Nesogale talazaci*, *Echinops telfairi*, *Tenrec ecaudatus*). Available TOGA-based annotation data from the armadillo (*Dasypus novemcinctus*) were added as an outgroup for the species tree reconstruction.

Phylogenetic analyses were based on amino acid alignments as they tend to be more conserved and less prone to systematic errors on deeper divergence cases [60, 61]. To generate amino acid alignments as the basis for phylogenetic reconstruction, we filtered the human reference-aligned amino acid sequences produced by TOGA. We used the generated orthology classification to filter the transcripts based on genes that were classified as one to one (1:1) orthologs between the reference human genome and the query species genome. Given that even one to one orthologs can have multiple ortholog chains, we filtered for duplicated transcripts based on sequence length and prevalence of gaps. Next, we filtered for genes that occurred at least once per species, obtaining a list of 12,062 genes. This list of genes was then used to filter transcripts in order to create one alignment per gene for the 14 chosen species using MAFFT v7.5 [62]. These alignments were then trimmed using ClipKit v2.3 [63], in kpi-smart-gap mode to dynamically determine the threshold of gaps to remove and keep parsimony informative sites only. Subsequently, the resulting alignments were filtered by sequence length keeping only those that were > 50 amino acids in length, thus obtaining a new filtered dataset corresponding to 7119 genes that are all over 50 parsimony informative sites in length.

Individual, unrooted maximum-likelihood gene trees for each of the 7119 amino acid alignments were generated using IQ-TREE v2.1.4 [64]. A Q.mammal + F+R9 substitution model was used for calculating each gene tree, with independently calculated log likelihood parameters. To account for anomalous long branches due to the estimation of an unrooted topology, we used TreeShrink v1.3.9 [65] to filter out outlier tip branches. A coalescence-based species tree was generated using ASTRAL v5.7.8 [66]. The resulting topology (without outgroup) was then used as the fixed input tree for MCMCTree in the PAML suite [67] for divergence time calculation. The R package MCMCTreeR [68] was used to prepare the input files and node age priors for the MCMCTree divergence time inference analysis. Fossil calibration estimates were designated using the oldest known fossil proving the existence of the branch and multiplying the hard minimum age by 1.25 to estimate a soft maximum age [69, 70] (Table 1). In line with Heritage et al., 2021 [71], age constraint intervals were placed on eight nodes representing crucial clade divergences within Afrotheria as follows: We used the *Ocepeia daouiensis* fossil (Node A; hard minimum at 60.5 Mya) to constrain the Paenungulata-Afroinsectiphilia split and thus as the oldest calibration for this dataset, this being the earliest known afrotherian fossil. *Eritherium azzouzorum* (Node B; 59 Mya) was chosen to calibrate the Paenungulata node; *Priscosiren atlantica* (Node D; 28 Mya) for the split between Dugongidae and Trichechidae within Sirenia; *Daouitherium rebouli* (Node E; 56 Mya) for the split between Proboscidea and Hyracoidea; *Heterohyrax auricampensis* (Node F; 10.4 Mya) for the divergence within Hyracoidea; and the earliest *Loxodonta sp.* (Node G; 6.5 Mya) for the split between extant proboscideans. Finally, within Afroinsectiphilia, *Todralestes variabilis* (Node C; 56.8 Mya) was used to constrain the split between Afrosoricida and Macroscelidea; *Oligorhynchocyon songwensis* (Node H; 25.2 Mya) for the split between Rhynchocyonidae and Macroscelididae within Macroscelidea.

**Table 1:** Fossil calibration ages used for divergence time inference within Afrotheria. All ages are displayed in the Mya (Million years ago) unit

Species	Hard minimum age (Mya)	Soft maximum age (Mya)	Node
*Ocepeia*	60.5	75.625	(A)
*Eritherium*	59	73.75	(B)
*Todralestes*	56.8	71	(C)
*Priscosiren*	28	35	(D)
*Daouitherium*	56	70	(E)
*Heterohyrax auricampensis*	10.4	13	(F)
*Loxodonta sp.*	6.5	8.125	(G)
*Oligorhynchocyon*	25.2	31.5	(H)

The approximate likelihood calculation for protein data function of MCMCTree v4.10.0 [72] was used in order to calculate divergence times within Afrotheria. Two datasets were created for this; a concatenated set of filtered gene trees that share the proposed coalescence-based topology of the species tree produced with ASTRAL and the concatenated set of all gene trees. IQtree’s QMaker function [73] was used to calculate amino acid substitution models for each dataset. Having specified the amino acid models, MCMCTree then uses CODEML [74] to calculate the Hessian matrix for protein data. Later, a Markov Chain Monte Carlo (MCMC) approach was used to calculate divergence times estimations using 1 million runs with 200 thousand burnin for both datasets. Multiple MCMCTree runs were performed and multiple dating scenarios were tested to check for convergence and effective sample sizes with Tracer v1.7.2 [75]. Estimates for both the filtered and the complete dataset were then visualized with TreeViewer v2.2.0 [76].

### Synteny analysis

In order to gain insights into chromosome evolution among afrotherian mammals, we obtained all high-quality chromosome-level genome assemblies produced for Afrotheria (Table S2). Chromosomes synteny of two elephants (*Loxodonta africana*, *Elephas maximus*), two sea cows (*Dugong dugon*, *Trichechus manatus*), one hyrax (*Heterohyrax brucei*), one aardvark (*Orycteropus afer*) and one sengi (*Rhynchocyon petersi*) was compared to our assembly of *Tenrec ecaudatus* chromosomes. Genome assemblies were manually curated to extract only autosomal chromosome scaffolds and the X sex chromosome scaffold, due to not having a male representative for all species. We used ntSynt v1.0.2 [77] to infer multi-genome synteny between all the available chromosome-level genome assemblies of afrotherian species and our new *Tenrec ecaudatus* genome. This program utilizes a minimizer graph-based approach, allowing for the calculation of multiple genome homologous synteny blocks with over 15% overall genome divergence using minimal resources. Furthermore, we used Mash v.2.3 [78] to calculate pairwise mutation distance and therefore, genome divergence between assemblies. For visualizing the synteny plots we used ntSynt-viz v1.0.0 [79].

## Results

### Genome assembly and annotation

We obtained a highly complete and contiguous haplotype-resolved genome assembly for the common tenrec, *T. ecaudatus*. (Table 2; Figure S1). Our primary haplotype was assembled to a total length of 4.06 Gb, whereas our secondary haplotype only had 3.44 Gb in length. Both reconstructed haplotypes showed remarkable BUSCO completion scores based on the set of conserved mammalian genes (Haplotype 1: mammalia odb10: C: 99.3% [S:96.92%, D:2.43%], F: 0.07%, M: 0.59%, n:9226; Haplotye 2: mammalia odb10: C: 97.16% [S:95.09%, D:2.07%], F: 0.11%, M: 2.73% n:9226). Added to this, the estimated k-mer based consensus quality (QV) portrays a very high base accuracy of 64.9 and 64.7 for both haplotypes respectively, indicating roughly one error per ~ 3.1 million base pairs. The discordance in quality and length observed between the assembled haplotype one and haplotype two can be associated to the latter consisting only of autosomal chromosomes. Considering the size of *T. ecaudatus* chromosomes, this adds up to a significant amount of the full genome assembly. Lastly, accumulated unassigned scaffolds being allocated mostly on haplotype one contribute further to this discordance in length. Taking all these factors into consideration, only haplotype one was considered on the quality metrics for comparative analyses. Repeated content amounted up to 62.8% of the genome assembly, with Long Interspersed Nuclear Elements (LINE) being the most represented group of repeated elements (31.02%) (Table S3).

**Table 2:** Primary and secondary haplotype assembly statistics of the *Tenrec ecaudatus* genome assembly

Assembly	Primary haplotype	Secondary haplotype
# contigs	5663	3611
Largest contig (mb)	294	295
Total length (Gb)	4.06	3.44
GC (%)	44.97	44.35
scaffold N50 (mb)	133.2	156.4
scaffold L50 (mb)	12	9
BUSCO completion	99.3%	95.09%
QV (Merqury)	64.9	64.7

**Fig. 2:**
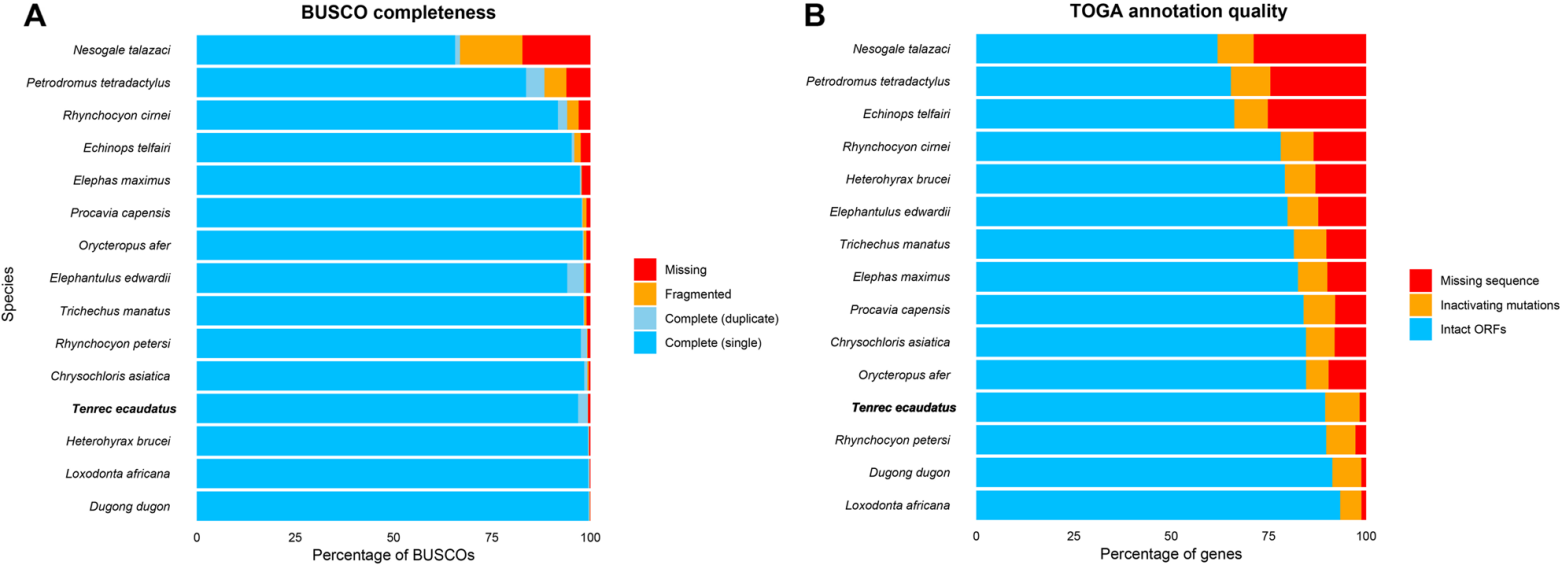
Quality metrics of all available genome assemblies of afrotherian mammals. **A** Shows the quality of the genome assembly on the percentage of BUSCO orthologous genes (%) that were classified as Complete, Fragmented and Missing. **B** Shows quality of the genome assembly and annotation in the percentage (%) of genes catalogued by TOGA as Intact, Inactivated or Missing

Along this orthology based quality measure, the annotation-based quality measure calculated with TOGA yielded a total count of 17,420 genes with intact open reading frames, 1720 genes with a type of inactivating mutation and 324 missing genes. Both these measures of quality positively highlight our *Tenrec ecaudatus* genome assembly and annotation compared to the available set of afrotherian mammal genome resources (Fig. 2). In this assemblage of genomes, the previous representation of Tenrecidae consisted only of the scaffold-level genome assemblies of *Nesogale talazaci* (BUSCO C: 66.7%; TOGA Intact ORF genes: 61.8%) and *Echinops telfairi* (BUSCO C: 95.9%; TOGA Intact ORF genes: 66.1%) (Table S4).

### Phylogenomic reconstruction and dating

**Fig. 3:**
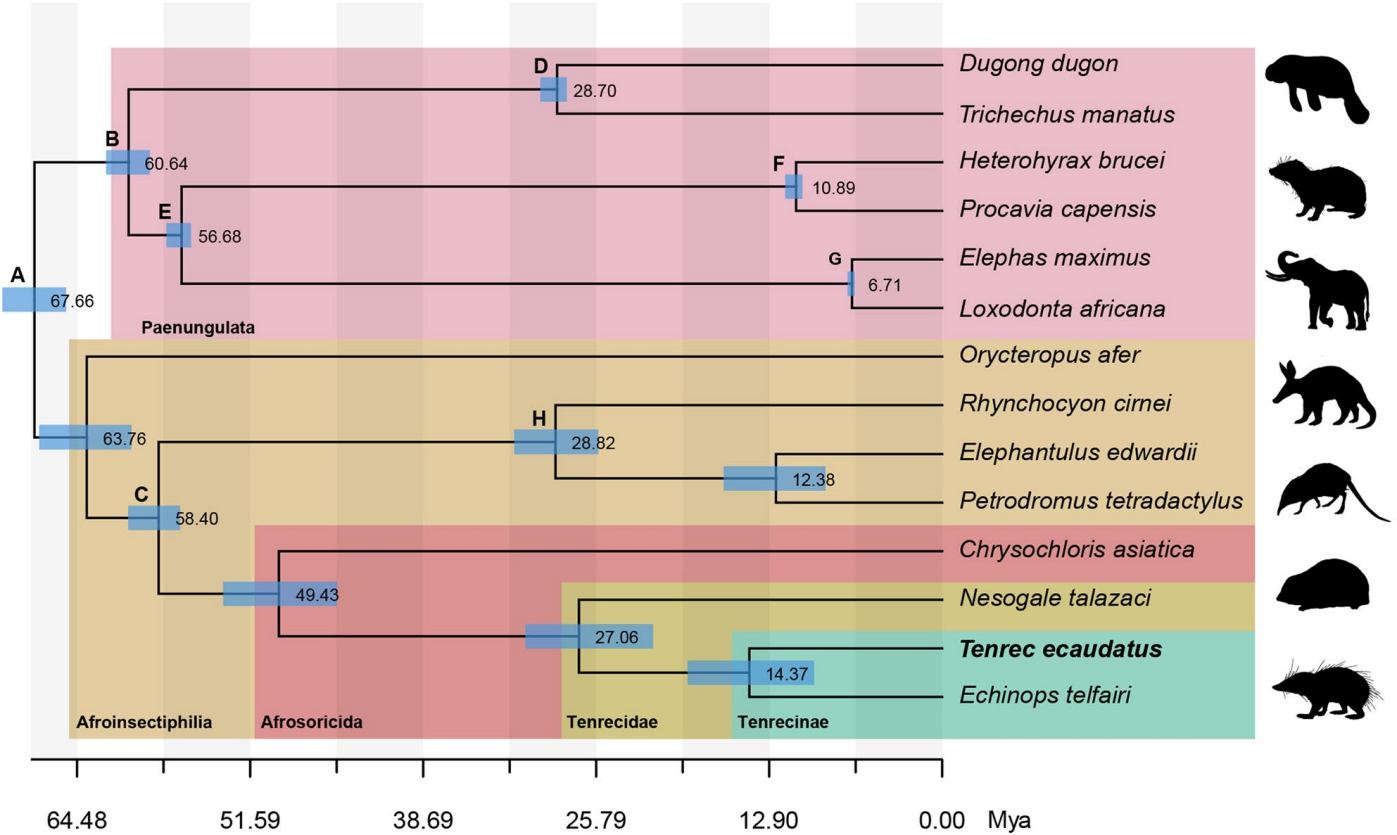
Phylogenetic reconstruction of Afrotheria and divergence time estimation using amino acid alignments. Fossil calibrated nodes are indicated alphabetically. Relevant clades mentioned are highlighted and colored by group. Blue bars indicate 95% Highest Posterior Density (HPD) of node ages. All nodes have posterior probability of 1 in the ASTRAL species tree

We reconstructed the phylogenetic relationships of tenrecs within Afrotheria (Fig. 3) using a protein alignment dataset for both the species with available annotation data and those with annotations generated in this study. The coalescence-based species tree estimated with ASTRAL obtained a posterior probability of 1 on each node (Figure S2). The newly sequenced *Tenrec ecaudatus* is placed as sister species to the lesser hedgehog tenrec (*Echinops telfairi*) within Tenrecinae (spiny tenrecs). Notably, our coalescent-based topology recovered Hyracoidea as a sister group to Proboscidea. The uncalibrated nodes within Afroinsectiphilia estimate the split of tenrecs from golden moles at around 49.4 (95%HPD=[45.105, 53.5972]) Mya. The following split of Tenrecinae from other tenrecs is estimated to 27.06 (95%HPD=[21.5568, 31.0638]) Mya. and the divergence time between *T. ecaudatus* and *E. telfairi* is estimated at 14.37 (95%HPD=[9.55502, 18.9804]) Mya.

### Synteny analysis

**Fig. 4:**
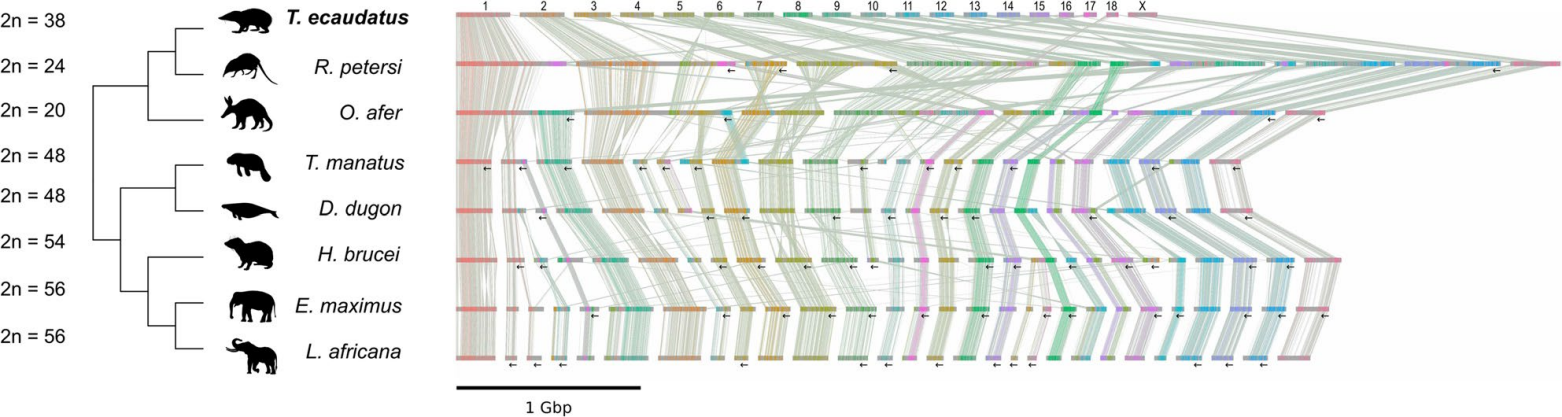
Ribbon plot representing chromosome synteny between chromosome-scale genome assemblies available for afrotherian mammals and aligned to the chromosomal scaffolds of *T. ecaudatus*. Chromosomes are represented by bars on a colour gradient, numbered at the top from larger to smaller autosomal scaffold for *T. ecaudatus*. X chromosomes are represented at the rightmost end. Black arrows represent chromosomal scaffolds whose orientation has been normalized to the target chromosomal scaffold of *T. ecaudatus*. A black line at the bottom of the plot represents a scale of 1 Gb

We inferred multi-genome synteny for eight chromosome-level genome assemblies of afrotherian species including our novel *Tenrec ecaudatus*, representing all six orders within the clade (Fig. 4). All inferred chromosomes were aligned based on synteny with *T. ecaudatus*. The first synteny blocks corresponding with Chr. 1–2 in *T. ecaudatus* show to be highly conserved between species. Though Afroinsectiphilia in particular showed remarkably high chromosome divergence.

Between the common tenrec (*T. ecaudatus*) and the giant sengi (*R. petersi*), there is a complex display of chromosome rearrangements including fission/fusion events on all chromosomes as well as multiple instances of inversions. Similarly, several inversions and fission/fusion events can be found between the chromosomes of *R. petersi* and *O. afer*, most notably, the event that resulted in Chr. 4 of the latter with one half corresponding to the inverted Chr. 10 of *R. petersi* and the other half corresponding to the inverted Chr. 4 of the same species. In the case of Paenungulata, the compared representatives show a much more conserved chromosome synteny than their sister clade. Both sea cow species show the same chromosome number, yet with a different chromosome configuration. Moreover, both representatives of extant Proboscidea show full microsynteny, while differing in overall chromosome scaffold length after chromosome 13.

## Discussion

We here present two high-quality haplotypes of the genome of *T. ecaudatus*, plus its comprehensive annotation. Although commonly used as an indicator of genome quality, the scaffold N50 metric does not directly link to better quality of genome assembly [80] in this particular phylogenetic order-wide comparison, due to the highly variable chromosome number and genome sizes across Afrotheria [81]. Such is the case of the aardvark assembly, showing a highly superior N50 metric (644 Mb) due to its massive chromosomes, and therefore, scaffold sizes. In cases like this where the compared assemblies are of outstanding quality and mostly at a chromosome-scale, BUSCO [82] completion can be a more appropriate metric of assembly quality.

Afrotherian genomes are among the largest ones among mammals [41], which has been attributed to their high concentration of repeated elements [83–85]. Our *T. ecaudatus* genome assembly and annotation aligns with this precedent, as 62.82% of the total genome content of haplotype 1 consisted of repeated elements (Table S3), mostly represented by the LINE type (31.02%) and simple repeats (17.61%). Currently, Afrotheria is not well represented genomically. Despite being a diverse superorder, only a fraction of afrotherian species have an available high-quality genome assembly. Out of the now 16 species that have an available genome assembly, our *Tenrec ecaudatus* assembly ranks as the highest quality assembled and annotated one for Tenrecidae and Afrosoricida, while also qualifying as one of the top five highest quality genomes for Afrotheria in terms of annotation and BUSCO completion (Fig. 2; Table S4). High-quality annotation can be attributed to the use of multiple-tissue transcriptome data to complement the TOGA produced annotation [56, 86].

Using eight fossil calibration points, our results are consistent with modern phylogenetic inference approaches that recover Afroinsectiphilia as a monophyletic clade [12, 87, 88]. It supports a basal split within Afrotheria between Afroinsectiphilia and Paenungulata and recovered hyraxes as a sister group to elephants, thus rejecting the Tethytheria hypothesis (elephants + sea cows) [89, 90]. These calibrations corresponded to the oldest available fossil for a determined group and estimated soft maximum bounds. Since including multiple calibrations, the determination of the upper bound is not as key of a factor as when using a single calibration point [70]. Accordingly, calibrated nodes showed small confidence intervals around the estimated hard minimum and soft maximum ages. Though, nodes that remained uncalibrated, such as the ones within Afrosoricida showed broader intervals of confidence. The afrosoricidan fossil record is very limited and fragmented, and the phylogenetic position of most extinct species remains unclear [15]. Thus, these nodes currently lack any fossil-informed calibration that could help increase the precision of temporal estimates [71]. In our estimates, the nodes comprising Afrosoricida (95%HPD=[45.105, 53.5972] Mya), Tenrecidae (95%HPD=[21.5568, 31.0638] Mya) and Tenrecinae (95%HPD=[9.55502, 18.9804] Mya), show younger mean divergence times than in Everson et al. (2016), which estimates a mean divergence of 58.4 Mya for Afrosoricida, 35.5 Mya for Tenrecidae and 25.7 Mya for Tenrecinae. Our results further show that even with a genome-wide dataset of phylogenetic data, divergence dating can be challenging for some nodes if there is no uniform temporal signal among genes. The choice of the clock model, too, has recently been shown to affect divergence dating within afrotherians when ingroup fossils for calibration are lacking [69]. We recommend future work to focus on this issue of divergence dating within afrosoricidans as particularly the split within Tenrecidae can be indicative of the minimum colonization date of Madagascar.

Tenrecidae is also currently the family among Afrotheria with the least amount of cytogenetic studies [41]. In terms of currently known chromosome numbers, afrosoricidans range from 2n = 26 to 2n = 34 in golden moles [40, 91, 92], keeping highly conserved chromosome numbers, whereas in Tenrecidae chromosome numbers are more variable. This variation ranges from 2n = 30 (or even from 2n = 14 according to an unsupported claim of [40]) to 2n = 56 [41, 93]. Therefore, our chromosome number estimation ranges within this reported variation.

Although some studies involving chromosome painting proposed the aardvark’s karyotype to share a strong resemblance with the eutherian ancestral state [94], more recent studies on mammal chromosome evolution hypothesize that the last common ancestor (LCA) of all placental mammals possessed 19 autosomal pairs (2n = 40) and showed great chromosomal conservation with the LCA of Amniota. Similarly, the hypothesized ancestral karyotype for Atlantogenata (Xenarthra + Afrotheria) would have shown the same number of chromosomes, differing only by four chromosome inversions accumulated over 5 million years [95]. Thus, chromosome number in *T. ecaudatus* is closer to the ancestral state in placental mammals and potentially afrotherians as well.

Several million years of evolution have resulted in Afrotheria displaying great chromosome variation between orders, meanwhile maintaining a high degree of synteny for the sex chromosomes, which has been very well documented in mammals [94, 96–98]. This can be seen in the chromosome rearrangements observed between the common tenrec (*T. ecaudatus*), giant sengi (*R. petersi*) and aardvark (*O. afer*), which despite varying a great amount in chromosome number, size and arrangement, still maintain conserved X chromosome synteny. In contrast to this highly variation within Afroinsectiphilia, elephant species *E. maximus* and *L. africana*, having had their divergence event much closer to present times, show virtually no chromosome rearrangements. The west indian manatee (*T. manatus*) and dugong (*D. dugon*) do not show the same level of conserved chromosome synteny as the compared elephant species, showing multiple chromosome reorganization events converging on the same chromosome number (2n = 48) [99]. Interestingly, *Trichechus inunguis*, a particular manatee species not included in this study, displays a karyotype of 2n = 56, eight more autosomes than its sister species *T. manatus*. These species of manatee have been reported to have diverged via chromosomal fusion events, a pericentric inversion and six Robertsonian translocations [100]. In other words, *D. dugon* and *T. manatus* exhibit the derived karyotypic state, whereas *T. inunguis* retains the ancestral state. This is further supported by Hyracoidea being hypothesized as having 2n = 54 as their ancestral state, which *H. brucei* and other hyrax species maintain until today [101]. This same species also shows very few chromosome rearrangements that separate them from proboscideans, mainly an inversion on Chr. 19 and fission/fusion of Chr. 9 and 10 into three and two separate chromosomes respectively. Future studies on this topic should use all these newly available chromosome-level genome assemblies to infer the ancestral karyotype of the last common ancestor of placental mammals.

Overall, the high-quality genomic resource generated in this study for *T. ecaudatus* is a valuable complement for genomic analyses such as phylogenomic reconstruction, multi-genome chromosome synteny inference and, although challenging, molecular divergence dating. These insights gained into the evolutionary history of *T. ecaudatus* and Afrosoricida are just a first example of the potential that genomics offers to bridge the gap in the knowledge of tenrec evolutionary history. Further analyses should focus more on delving into the plesiomorphic characters present in *T. ecaudatus* physiology, reproduction and development, and their genomic basis in order to explore how these make tenrecs unique creatures and how mammalian genomes may have evolved through the ages.

## Supplementary Information

Supplementary Material 1.

## Data Availability

The genome annotation data newly generated with TOGA for *Tenrec ecaudatus* and the annotation data generated previously are available at: https://genome.senckenberg.de/download/TOGA/human\_hg38\_reference/Afrotheria/. Raw data and genome assembly data have been deposited in NCBI under BioProject accession number PRJNA1359382.

## Abbreviations

Mya: Million years ago
IUCN: International Union for Conservation of Nature
DEPC: Diethyl pyrocarbonate
ng: Nanograms
PacBio: Pacific Biosciences
bp: Base pairs
BUSCO: Benchmarking universal single-copy orthologues
Gb: Giga base-pairs
MCMC: Markov Chain Monte Carlo
ORF: Open reading frame
Chr: Chromosome
HPD: Highest posterior density
LCA: Last common ancestor
LINE: Long Interspersed Nuclear Elements
