# Supplementary material for: Chromosome-level genome assembly of the common tenrec, Tenrec ecaudatus (Schreber, 1778), a new model for early placental mammal evolution
Source: BMC Genomics. 2026 Mar 31;27:365. doi: 10.1186/s12864-026-12794-9 (PMC13063843; doi:10.1186/s12864-026-12794-9)
Supplement: Supplementary file 1 — Supplementary Material 1. [file 12864_2026_12794_MOESM1_ESM.docx]

Supplementary Material:


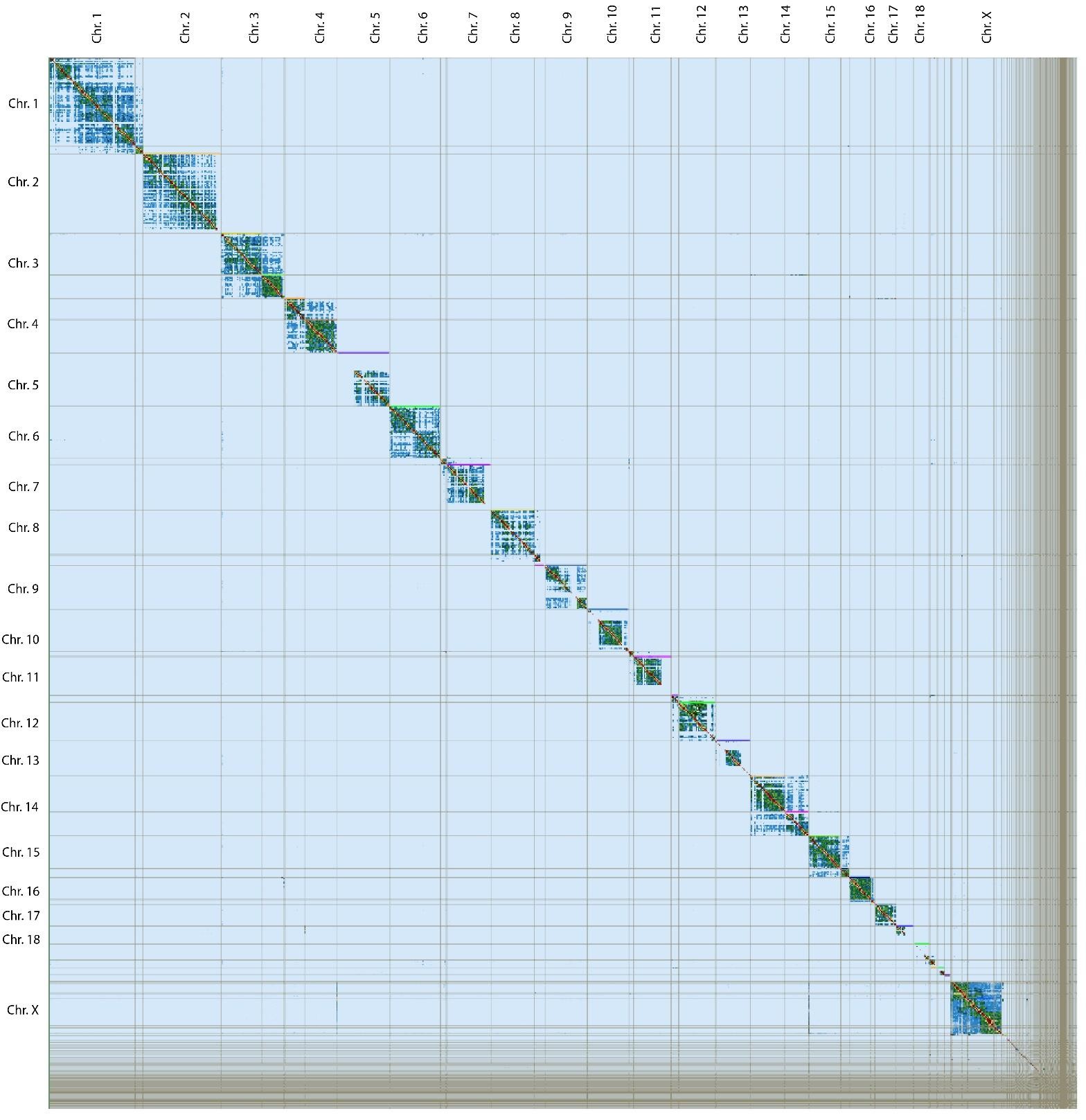
Figure S1: Hi-C heatmap displaying the contact density between genomic regions. The 18 assembled autosomal chromosomes (2n=38) are arranged by size. Sex chromosome X is shown at the bottom rightmost end of the plot.


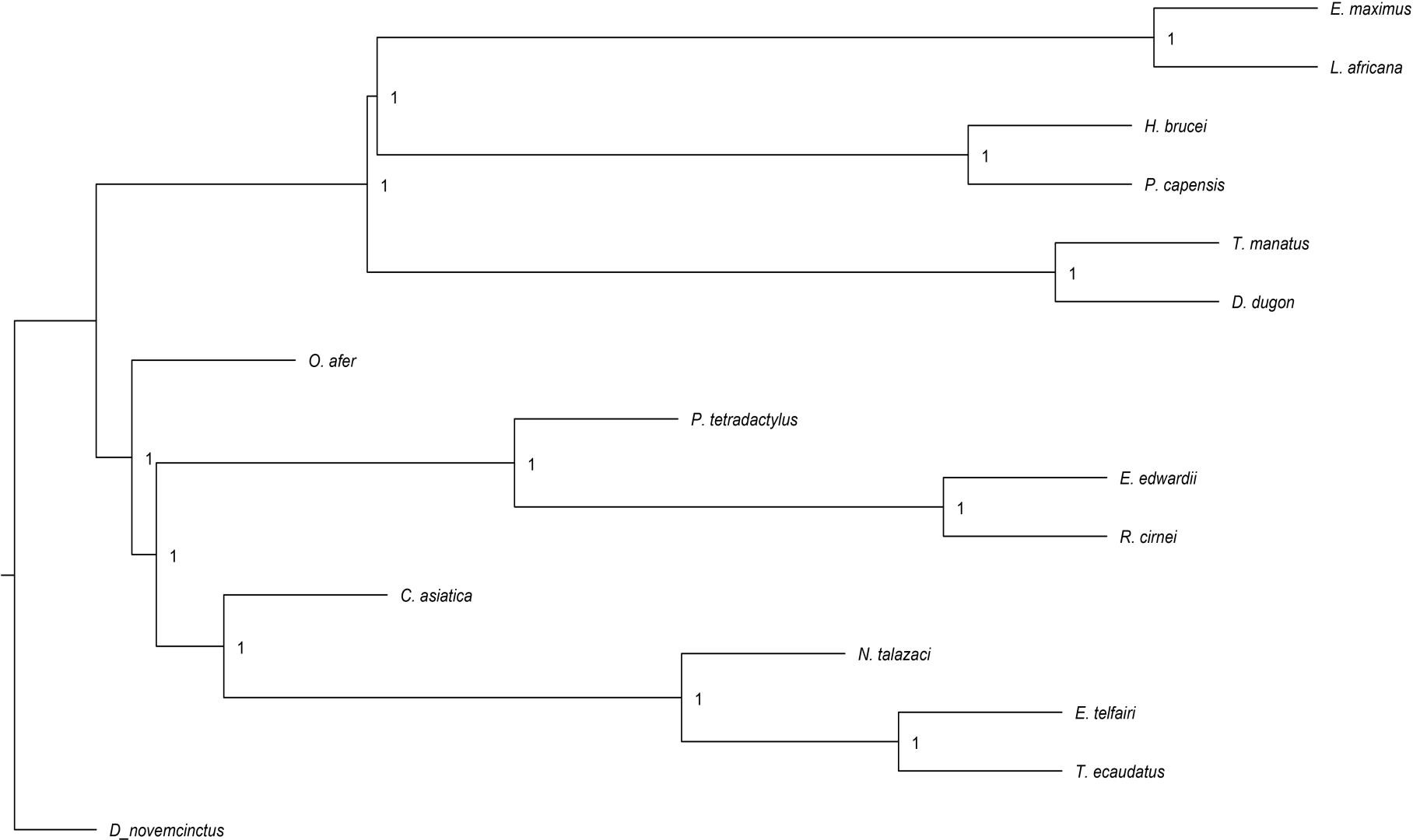
Figure S2: Phylogenetic reconstruction of Afrotheria using a coalescence-based approach. *D. novemcinctus* is used as an outgroup to Afrotheria. All nodes have posterior probability of 1.

Table S1: Sampled *T. ecaudatus* individuals’ respective ID’s, sex, activity state and extracted tissues.

| **ID #** | **Animal #** | **Fauna Sample ID** | **Sex** | **State** | **Tissue** |
| --- | --- | --- | --- | --- | --- |
| TE-103 | M20371 | TE103H | Male | Active 28/28 | Heart |
| - | TE-133-Embryo1 | 133-1E | - | - | Embryo |
| - | TE-135-Embryo1 | 135-1E | - | - | Embryo |
| TE-113 | F20376 | TE113H, TE113B, TE113K, TE113L | Female | Hibernating 12/12 | Heart, Brain, Kidney, Liver |
| TE-72 | M17806 | TE113TS | Male | Hibernating 12/12 | Testes |
| TE-98 | M20577 | TE98B, TE98K, TE98L, TE98TS | Male | Active 28/28 | Brain, Kidney, Liver, Testes |

Table S2: Species used for phylogenetic reconstruction and synteny analysis, including the origin of the assembly and the annotation data.

| **Species name** | **Assembly** | **TOGA annotation data** |
| --- | --- | --- |
| *Dugong dugon* | mDugDug1 (GCA_030035585.1) | This study |
| *Trichechus manatus* | HLtriManLat2 (DNAZoo) | Senckenberg Database |
| *Loxodonta africana* | mLoxAfr1 (GCF_030014295.1) | This study |
| *Elephas maximus* | mEleMax1 (GCA_024166365.1) | This study |
| *Heterohyrax brucei* | HLhetBru1 (GCA_004026845.1) | Senckenberg Database |
| *Procavia capensis* | HLproCap4 (DNAZoo) | Senckenberg Database |
| *Orycteropus afer* | OryAfe1.0_HiC (DNAZoo) | This study |
| *Chrysochloris asiatica* | chrAsi1 (GCF_000296735.1) | Senckenberg Database |
| *Rhyncocyon cirnei* | PRJNA1039701 (BioProject) | (Hagemann et al. Unp.) |
| *Elephantulus edwardii* | eleEdw1 (GCF_000299155.1) | Senckenberg Database |
| *Petrodromus tetradactylus* | PRJNA1039702 (BioProject) | (Hagemann et al. Unp) |
| *Nesogale talazaci* | HLmicTal1 (GCA_004026705.1) | Senckenberg Database |
| *Echinops telfairi* | echTel2 (GCF_000313985.1) | Senckenberg Database |
| *Tenrec ecaudatus* | This study | This study |
| *Dasypus novemcinctus* | dasNov3 (GCF_000208655.1) | Senckenberg Database |

**Senckenberg database for TOGA annotations available at: https://genome.senckenberg.de/download/TOGA/human_hg38_reference/Afrotheria/*

Table S3: Repeated elements detected on haplotype 1 of the assembled *T. ecaudatus* genome.

| **Type of repeat** | **Percentage** | **# bases** |
| --- | --- | --- |
| SINE | 4.236% | 172404236 |
| LINE | 31.022% | 1262479008 |
| LTR | 6.783% | 276061505 |
| DNA | 4.198% | 170847344 |
| Simple | 17.613% | 716765757 |
| Other | 7.801% | 317462267 |
| TOTAL | 62.829% | 2556908948 |

Table S4: Genome assembly quality metrics based on contiguity and length of all available afrotherian mammal assemblies used in this study.

| **Species name** | **Assembly** | **Assembly level** | **Chromosome #** | **Total length (Gb)** | **Scaffold N50 (Mb)** | **Scaffold L50** | **BUSCO**  **Completion (%)** |
| --- | --- | --- | --- | --- | --- | --- | --- |
| *Dugong dugon* | mDugDug1 (GCA_030035585.1) | Chromosome | 2n=48 | 3.2 | 140.7 | 9 | 99.7 |
| *Trichechus*  *manatus* | HLtriManLat2 (DNAZoo) | Chromosome | 2n=56 | 3.1 | 143.7 | 9 | 98.4 |
| *Loxodonta*  *africana* | mLoxAfr1 (GCF_030014295.1) | Chromosome | 2n=56 | 3.5 | 119.6 | 11 | 99.8 |
| *Elephas maximus* | mEleMax1 (GCA_024166365.1) | Chromosome | 2n=56 | 3.4 | 127.4 | 10 | 97.7 |
| *Heterohyrax brucei* | HLhetBru1 (GCA_004026845.1) | Chromosome | 2n=54 | 3.5 | 139.6 | 10 | 99.6 |
| *Procavia capensis* | HLproCap4 (DNAZoo) | Scaffold | 2n=54 | 3.6 | 133.7 | 11 | 98 |
| *Orycteropus afer* | OryAfe1.0_HiC (DNAZoo) | Chromosome | 2n=20 | 4.2 | 644 | 3 | 98.2 |
| *Chrysochloris*  *asiatica* | chrAsi1 (GCF_000296735.1) | Scaffold | 2n=30 | 4.2 | 13.5 | 85 | 99.2 |
| *Rhyncocyon cirnei* | PRJNA1039701  (BioProject) | Scaffold | Unknown | 5.4 | 0.7 | 1926 | 94.1 |
| *Elephantulus*  *edwardii* | eleEdw1 (GCF_000299155.1) | Scaffold | 2n=26 | 3.8 | 15 | 83 | 98.3 |
| *Petrodromus*  *tetradactylus* | PRJNA1039702  (BioProject) | Scafffold | 2n=28 | 3.9 | 0.5 | 2167 | 88.2 |
| *Nesogale talazaci* | HLmicTal1 (GCA_004026705.1) | Scaffold | 2n=30 | 3.2 | 0.065 | 14134 | 66.7 |
| *Echinops telfairi* | echTel2 (GCF_000313985.1) | Scaffold | 2n=40 | 2.9 | 54 | 17 | 95.9 |
| *Tenrec ecaudatus* | This study | Chromosome | 2n=38 | 4.06 | 133.2 | 12 | 99.3 |
| *Rhynchocyon petersi* | mRhyPet1 (GCA_043290085.1) | Chromosome | 2n=24 | 5.6 | 522 | 5 | 99.1 |
